# Recurrent Pediatric Extrapleural Solitary Fibrous Tumor of the Jaw

**DOI:** 10.1007/s12105-025-01816-9

**Published:** 2025-07-29

**Authors:** Baishakhi Modak, Vishwapriya Mahadev Godkhindi, Narayana Venkadasalapathy, Raghu Radhakrishnan

**Affiliations:** 1 Department of Oral and Maxillofacial Pathology, Manipal College of Dental Sciences, Manipal Academy of Higher Education, Manipal, Karnataka 576104 India; 2 Mangalore Institute of Oncology, Mangaluru, Karnataka 575007 India; 3 Hannah Joseph Hospital Pvt. Ltd. Chintamani, Madurai, Tamil Nadu 625 009 India; 4 Unit of Oral Biology and Oral Pathology, Oman Dental College, 617 Way, Muscat 116, Wattayah, Sultanate of Oman

**Keywords:** Case report, Solitary fibrous tumor, Pediatric, STAT6 transcription factor, Cell differentiation, Pleural neoplasms

## Abstract

**Background:**

Solitary fibrous tumors encompass a heterogeneous group of spindle cell neoplasms, ranging from biologically low-risk lesions to, in rare instances, highly aggressive tumors with malignant potential. Dedifferentiation in solitary fibrous tumors is uncommon and typically occurs in the retroperitoneum, with extrapleural involvement being among the least frequently reported.

**Case Presentation:**

A 13-year-old male presented with a rapidly enlarging mass in the lower jaw of 20 days duration, involving the submandibular triangle and floor of the mouth.

**Diagnosis:**

Histopathological examination of the excisional biopsy revealed spindle-shaped cells arranged in compact fascicles with a haphazard distribution, and areas of hyalinization. Immunohistochemical analysis demonstrated positivity for CD34, STAT6, MyoD1, α-SMA, Bcl-2, and CD99, confirming the diagnosis of extrapleural dedifferentiated solitary fibrous tumor (DSFT).

**Management:**

The lesion was surgically excised but recurred, likely due to disease progression. Re-excision was planned, but the child died 10 days before surgery.

A 13-year-old male was referred with a rapidly enlarging mass in the lower jaw, present for 20 days. The chief complaint concerned an intraoral mass causing lateral displacement of the tongue, along with difficulty in swallowing, breathing, speaking, and intraoral bleeding. Intraorally, a well-defined mass involving the submandibular triangle and floor of the mouth was noted, obliterating the vestibule and restricting tongue movement (Fig. 1). Radiographically, an orthopantomogram revealed a radiolucent lesion extending from FDI tooth 35 to the anterior border of the ramus, with thinning of the mandibular border (Fig. 2). Contrast-enhanced CT scan demonstrated a well-circumscribed ovoid lesion with expansion of the lingual cortical plate.

**Fig. 1:**
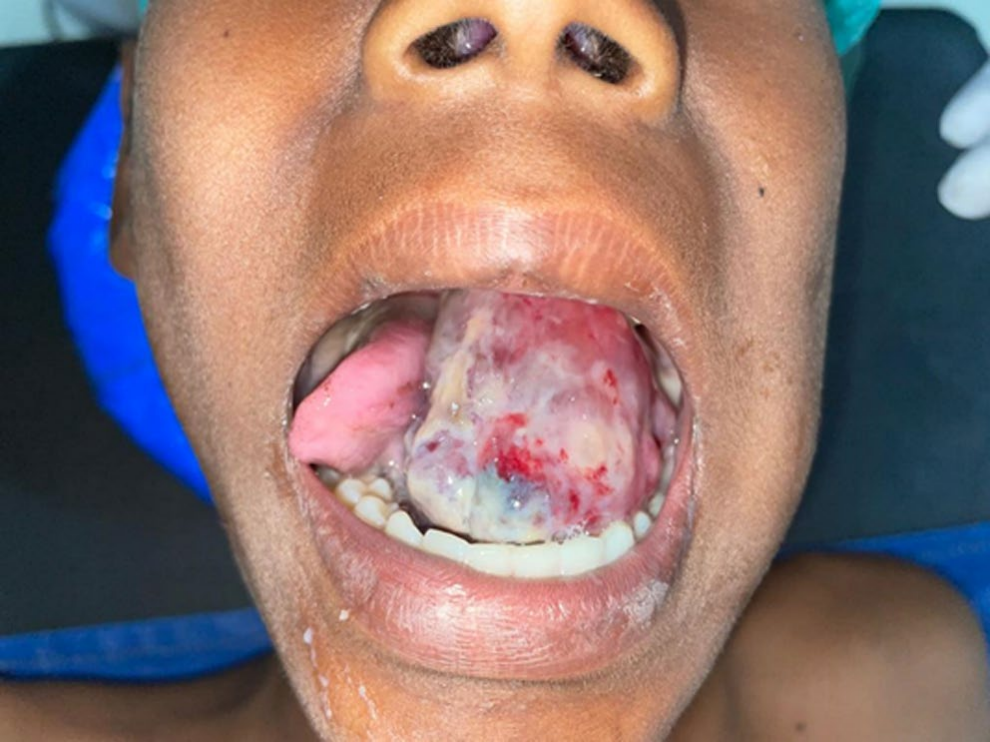
A rapidly enlarging mass situated in the submandibular triangle

**Fig. 2:**
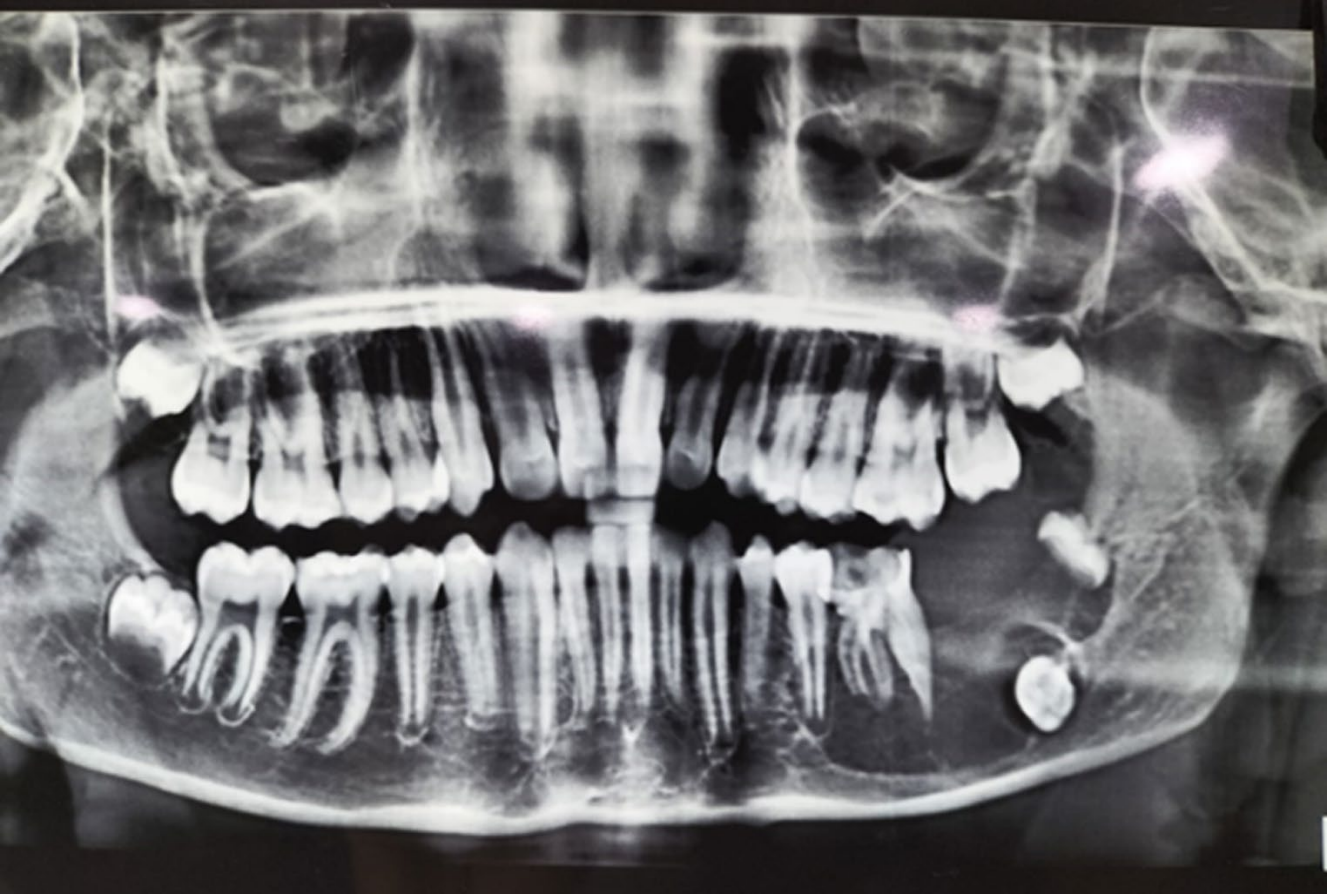
An orthopantomogram revealing a well-corticated radiolucent lesion located on the left side of the lower jaw

An excisional biopsy confirmed the diagnosis. Histopathological analysis revealed spindle-shaped cells (Fig. 3A) with a haphazard arrangement, mitotic activity, and areas of hyalinization (Fig. 3B]. The patient’s history of a rapidly tumor growth and intraoral bleeding prompted a differential diagnosis that included myofibroma, rhabdomyosarcoma, solitary fibrous tumor (SFT), angiosarcoma, and synovial sarcoma. Immunohistochemistry showed diffuse positivity for α-SMA and focal nuclear positivity for STAT6 (Fig. 4A), Bcl-2 (Fig. 4B), and CD99 (Fig. 4C), with absence of nuclear or membranous staining for MyoD1 and beta-catenin, and no staining for CD34. Based on review of differential diagnoses, the lesion was diagnosed as dedifferentiated solitary fibrous tumor (DSFT).

**Fig. 3:**
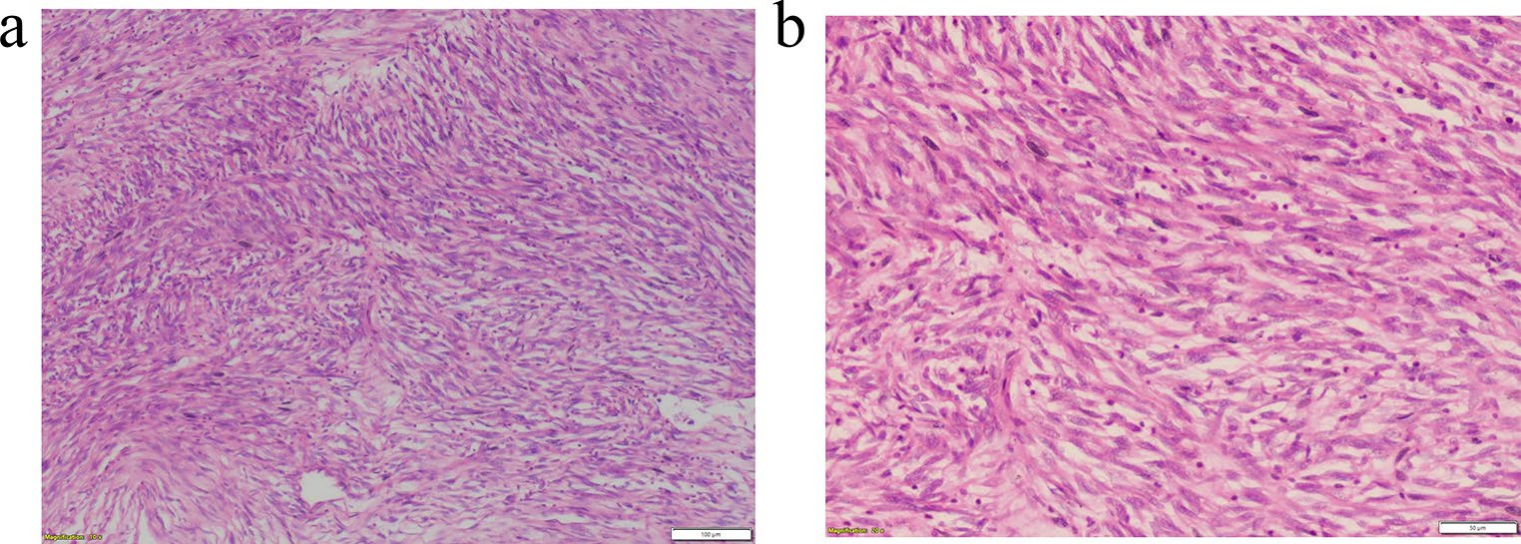
Hematoxylin and eosin-stained section showing (**A**) a cellular tumor composed of spindle-shaped cells arranged in compact fascicles within a collagen-rich stroma (10×, H&E); (**B**) haphazard neoplastic spindle cells with nuclear pleomorphism, mitoses, and multinucleated giant cells (20×, H&E)

**Fig. 4:**
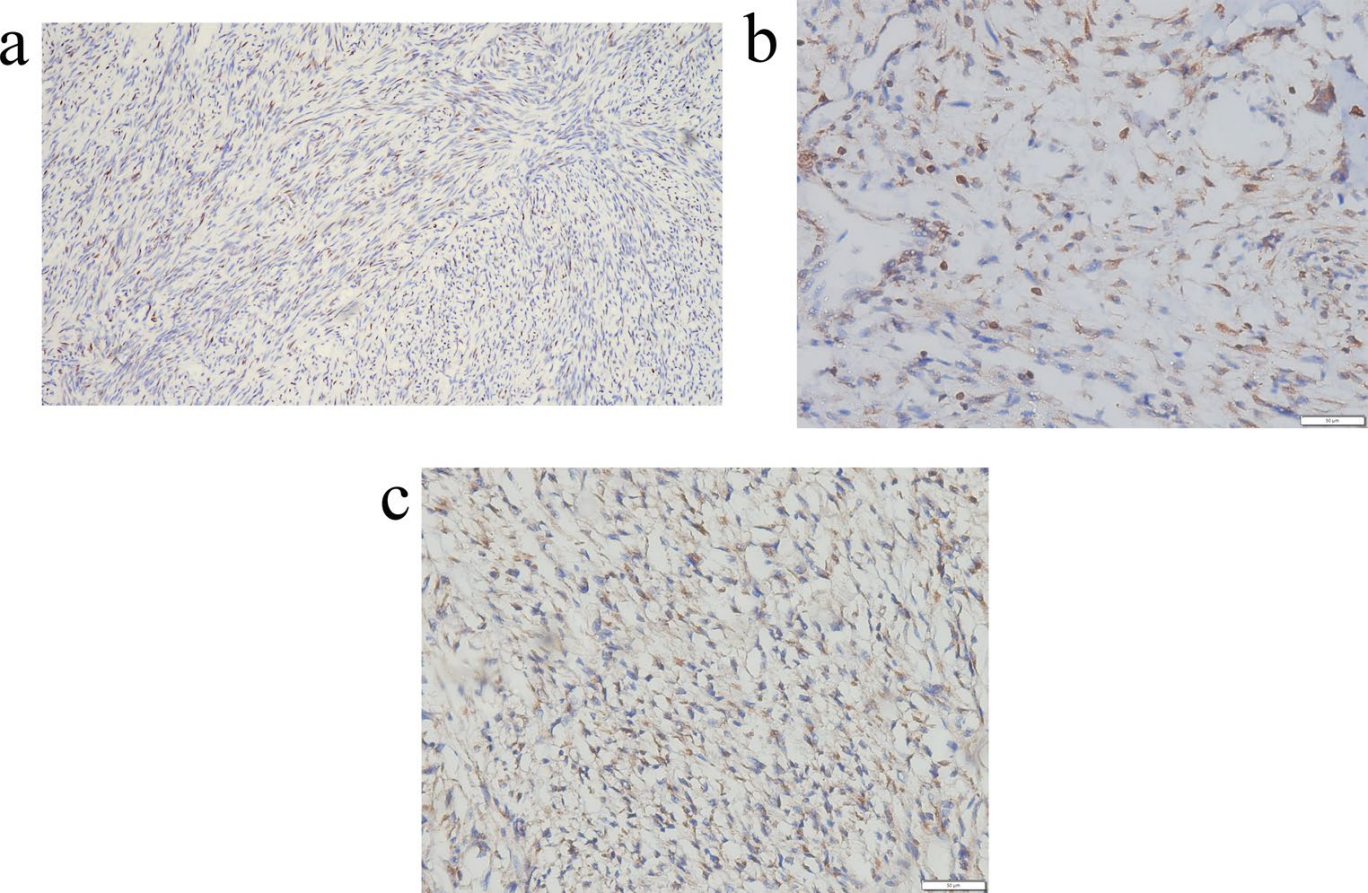
Immunohistochemical staining reveals (**A**) focal nuclear positivity for STAT6 (10×). (**B**) cytoplasmic positivity for Bcl2 in tumor cells (20×) and (**C**) limited nuclear positivity for CD99 in tumor cells (20×)

While CD34 serves as a reliable marker for diagnosing conventional SFTs, its positivity may be lower or absent in dedifferentiated cases. The detection of the NAB2-STAT6 fusion gene can aid in diagnosis, but availability may vary across laboratories. Immunohistochemistry for STAT6 offers an alternative method for detecting the fusion gene, with diffuse nuclear positivity typically observed in conventional SFTs.

The lesion was surgically excised, and the patient initially experienced a favorable postoperative outcome. However, the lesion recurred after two months, causing significant facial asymmetry with pulmonary metastases (Fig. 5). A CT angiogram demonstrated tumor expansion from the mandibular first premolar to the condyle and coronoid process on the left side, displaying with a network of interconnected blood vessels originating from the maxillary and facial arteries. Surgical re-excision was recommended, but unfortunately, the child died 10 days later prior to additional surgery.

**Fig. 5:**
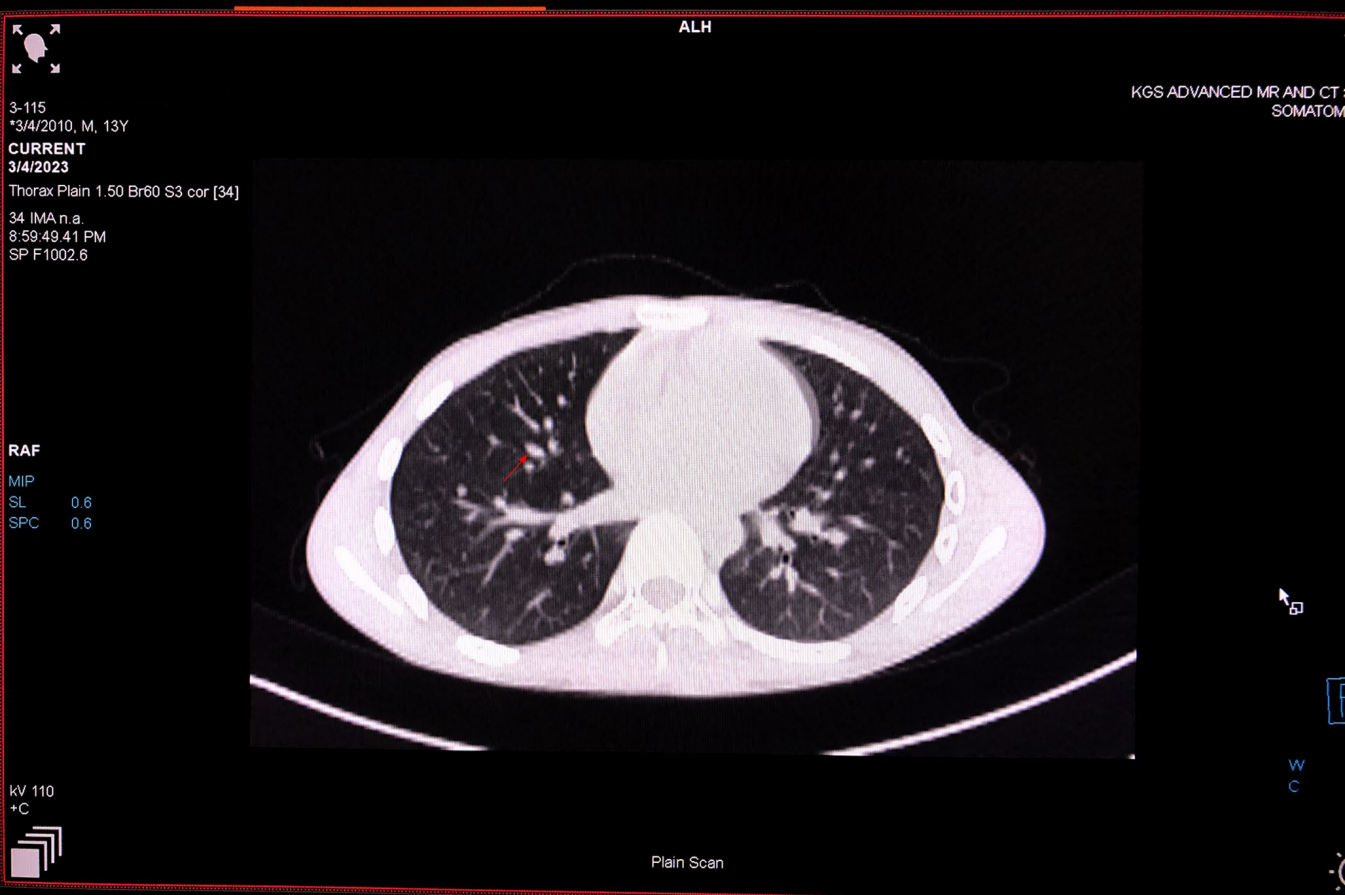
Recurrence of the mass observed two months after surgery, and a CT scan revealed multiple bilateral pulmonary nodules (red arrow) in the lung parenchyma

Solitary fibrous tumor (SFT), known for its borderline malignancy and unpredictable behaviour, often presents challenges in both diagnosis and management [1]. Clinically, SFTs in the oral cavity typically present as submucosal nodules with well-defined borders, often asymptomatic in the early stages [2]. Although dedifferentiation within SFT is rare, it has garnered increasing attention due to its distinct histopathological and clinical characteristics [3]. It may present with symptoms such as shortness of breath, pain, weight loss, and rapid growth, in contrast to the typically slow-growing, painless nature of conventional SFTs. While metastases commonly occur in the lung, reports have also documented spread to the brain, liver, and bones [4].

In the present case, focal positivity for STAT6, Bcl-2, and CD99 supported the diagnosis of dedifferentiated solitary fibrous tumor (DSFT). The distinction between conventional and dedifferentiated SFTs underscores the importance of accurate diagnosis. It further highlights the need for ongoing clinical surveillance and continued research to enhance our understanding of therapeutic strategies and prognostic factors associated with this rare malignancy. This represents the first reported case of extrapleural DSFT in a pediatric patient.

## Data Availability

No datasets were generated or analysed during the current study.
